# Mechanosensing of Shear Stress and Uterine Spiral Artery Remodeling by Invasive Trophoblasts in Early Pregnancy

**DOI:** 10.3390/ijms26199565

**Published:** 2025-09-30

**Authors:** Dariusz Szukiewicz, Seweryn Trojanowski, Edyta Wróbel, Piotr Wojdasiewicz, Grzegorz Szewczyk

**Affiliations:** 1 Department of Biophysics, Physiology & Pathophysiology, Faculty of Health Sciences, Medical University of Warsaw, 02-004 Warsaw, Poland; edyta.wrobel@wum.edu.pl (E.W.); piotr.wojdasiewicz@wum.edu.pl (P.W.); grzegorz.szewczyk@wum.edu.pl (G.S.); 2 Chair and Department of Obstetrics, Gynecology and Gynecological Oncology, Medical University of Warsaw, 03-242 Warsaw, Poland; seweryn.trojanowski@wum.edu.pl

**Keywords:** shear stress, mechanosensing, trophoblast invasion, extravillous trophoblast, uterine spiral arteries, vascular remodeling, placentation, placental angiogenesis, preeclampsia/eclampsia

## Abstract

The development of low-resistance blood flow within the developing placenta in the early weeks of pregnancy requires trophoblast invasion of the uterine spiral arteries. Therefore, understanding the migration and differentiation of trophoblasts is necessary. Recently, researchers have focused increasingly on the regulation of the response of endovascular extravillous trophoblasts (enEVTs) to mechanical stimuli associated with shear stress. The starting point for these studies is that enEVTs, which adopt a pseudoendothelial phenotype, functionally resemble endothelial cells in terms of ability to promote angiogenesis, vascular remodeling and cell–cell communication. The complex process of mechanotransduction requires the coordinated participation of many types of mechanoreceptors, whose activated signaling pathways are translated into whole-cell mechanosensing involving components of the cytoskeleton and extracellular matrix. The aim of this review is to comprehensively present the current knowledge on the importance of mechanical stimuli associated with shear stress in the development of local changes in the vascular system at the site of blastocyst implantation. The characteristics of individual mechanoreceptors are determined, and the most important factors influencing mechanotransduction are discussed. Understanding the importance of mechanosensing disorders in trophoblasts in the pathogenesis of unexplained recurrent abortions or preeclampsia may be helpful in the development of new therapeutic strategies based on the regulation of mechanotransduction in response to shear stress.

## 1. Introduction

In the closed circulatory system, which occurs in humans and all vertebrates, the flow of blood pumped by the heart, assisted by accessory mechanisms (i.e., arterial elasticity, venous valves, a muscular–venous pump, negative intrathoracic pressure, anastomosis and “up and down” movement of the atrio-ventricular plane of the heart), is essentially always unidirectional from the heart to the periphery and from the periphery back to the heart [1]. The force exerted by blood flow does not act perpendicular to the surface of the vessel wall but rather parallel to the vascular endothelial cells, contributing to the generation of shear stress [2]. Shear stress perceived within the physiological range and within a specific time frame by endothelial cell mechanoreceptors is an important stimulus that regulates many processes, including cell migration, alignment and polarity, barrier function, vesicular transport, permeability, renal transport, fluid homeostasis, short-term vasoreactivity, vascular morphogenesis, and long-term remodeling [2,3,4,5,6,7]. Moreover, physiological shear stress increases the viability of endothelial cells by strongly inhibiting their apoptosis, which results from, among other factors, the stimulation of protein kinase B (PKB, also known as Akt) phosphorylation [8,9]. However, both excessive destructive shear stress and markedly reduced shear stress (e.g., in the popliteal artery during prolonged sitting) can lead to endothelial dysfunction with a reduction in cell growth and viability, damaging them and increasing cell mortality, including through increased apoptosis [10,11,12,13,14]. On the clinical side, mechanotransduction disorders at the endothelial level caused by nonphysiological shear stress may lead to tissue perfusion disorders, including placental hypoperfusion and accelerated development of atherosclerotic plaque or aneurysm formation [15,16,17,18,19,20].

In human pregnancy, embryo implantation occurs at the blastocyst stage and key stages of implantation, such as contact (apposition), adhesion (attachment), and invasion (penetration), from 6 to 9–10 days after fertilization of the egg [21]. Good endometrial vascularization is crucial for the rapid growth and transformation of the blastocyst, which by the end of week 8 leads to the substantial formation of all the major tissues and structures of the new organism, ending the embryonic stage and beginning the fetal period [22,23]. The basic step in the formation of a low-resistance vascular bed, ensuring the supply of an adequate amount of oxygen and nutrients through blood to the developing placenta, is the penetration of the trophoblast into the spiral arteries of the uterus [24]. For this purpose, a single layer of trophoblasts surrounding the inner cell mass of the blastocyst differentiates into villous trophoblasts, which form the placental villi, and extravillous trophoblasts (EVTs) derived in humans from progenitor cells residing in extravillous columns, which are responsible for penetration into the uterus. Endovascular extravillous trophoblasts (enEVTs), a subtype of EVT, invade the wall of the spiral artery both interstitially and endovascularly and then induce endothelial cell apoptosis [25,26]. Despite occasional controversies, it is assumed that pseudovasculogenesis occurs during spiral artery remodeling, during which enEVTs completely and permanently replace endothelial cells and adopt a pseudoendothelial cell phenotype [26]. It follows from the above findings that the development of low-resistance and subsequently placental circulation during the first trimester of pregnancy is accompanied by a significant qualitative and probably quantitative change in the perception of shear stress, as endothelial cell mechanoreceptors are replaced by enEVT mechanoreceptors [27,28,29,30,31]. The current literature lacks synthetic studies on the role of shear stress in endothelial cell transformations and the differentiation and invasiveness of extravillous trophoblast cells related to human pregnancy.

The aim of this review is to comprehensively present the current knowledge on the importance of mechanical stimuli associated with shear stress in the development of local changes in the vascular system at the site of blastocyst implantation. Where possible, efforts have been made to limit the data to human pregnancy, and animal studies (mainly conducted in mice and rats) have been cited as supplementary data in the absence of human data, with full awareness of the potential differences.

The following topics will be discussed: shear stress and shear stress sensing, different types of mechanoreceptors and the phenomenon of mechanotransduction, also in the context of whole-cell mechanosensing. Next, shear stress will be discussed as an essential factor modulating spiral artery remodeling by regulating trophoblast invasiveness.

## 2. Shear Stress

In circulatory hemodynamics, the field of medicine relating to the principles that govern blood flow within the circulatory system, bloodstream shear stress is among the main parameters, as it denotes a mechanically generated force related to the friction exerted by blood flow on the inner lining of the blood vessel (endothelium) [32,33]. Shear stress, typically denoted by the Greek letter *τ* (tau), occurs when a force is applied parallel to a surface, causing it to deform by sliding or shearing. According to the International System of Units (SI), the measurement unit for shear stress is N/m^2^ or Pascal (1 Pa = 1 N/m^2^); less often, dyne per square centimeter (1 dyne/cm^2^ = 0.1 Pa) is used. It is estimated that under physiological conditions in humans, shear stress values in the venous system range from 0.1 N/m^2^ (1 dyne/cm^2^) to 0.6 N/m^2^ (6 dyne/cm^2^), whereas in arterial vessels, these values are at least 10 times greater and range from 1 N/m^2^ (10 dyne/cm^2^) to 7 N/m^2^ (70 dyne/cm^2^) [34,35].

Closely related to the concept of shear stress is another key concept in fluid mechanics, shear rate, which determines not the force but the rate at which a fluid deforms because of this force [36,37]. In other words, shear stress is the force that causes deformation, and shear rate is the measure of how quickly that deformation occurs. In a Newtonian fluid, the relationship between shear stress and shear rate is constant and linear, mainly because viscosity remains unchanged regardless of how quickly the fluid is flowing or being deformed [38]. Viscosity is the primary measure of a fluid’s resistance to flow or deformation under shear stress, and greater viscosity indicates greater resistance in the vascular system. Blood is a non-Newtonian fluid; its viscosity is not constant and changes depending on the shear rate [39,40,41,42]. Specifically, blood is a shear-thinning fluid, meaning that its viscosity decreases as the shear rate increases. The overall blood viscosity at a given moment consists of the concentration of erythrocytes (hematocrit), plasma viscosity, red blood cell deformability and the tendency of erythrocytes to form aggregates [43,44]. Moreover, blood viscosity is temperature dependent and usually decreases as the temperature increases but increases as the blood temperature decreases [45,46]. Slow blood flow (low shear rate), e.g., in small capillaries, causes an increase in blood viscosity, whereas an increase in the speed of the blood stream (higher shear rate), e.g., in larger arteries, is accompanied by a decrease in viscosity. This is because erythrocytes, which usually determine the viscosity of blood to the greatest extent, can move toward the center of the stream at increased flow speed and deform, reducing flow resistance [47]. The phenomenon of a decrease in apparent relative viscosity also applies to blood flow in small vessels with a diameter of less than approximately 0.3 mm, where it is known as the Fåhræus–Lindqvist effect or sigma effect [48].

**Figure 1:**
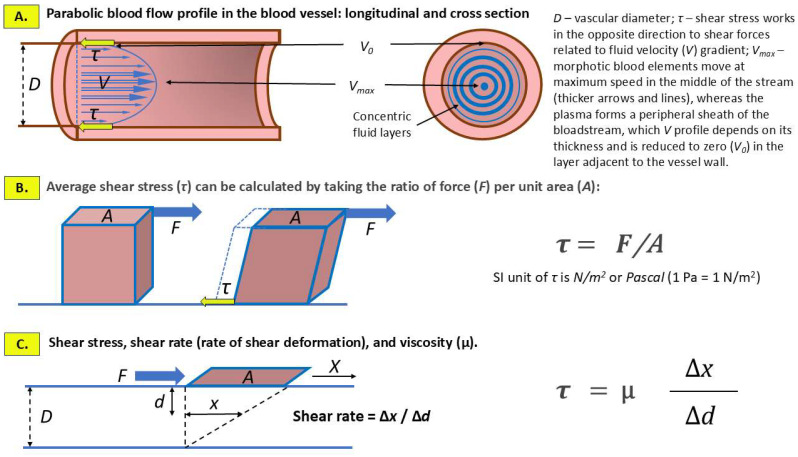
Hemodynamic shear stress and shear rate as a consequence of the frictional force exerted by blood stream on the inner lining of blood vessels including predominantly endothelial cells and, at the site of implantation, invading extravillous trophoblast. (**A**) Laminar (normal) blood flow is characterized by a parabolic velocity profile, which is caused by the variation in flow velocity, from the highest in the axis (center) of the vessel lumen (*V_max_*), gradually decreasing to zero (*V*_0_) towards the vascular wall. (**B**) SS is the tangential force exerted by blood flow on the cells covering the inner wall of the vessel. (**C**) Shear rate refers to the rate at which blood, considered as a non-Newtonian fluid, deforms due to the velocity gradient between adjacent layers of the blood stream. A plate (marked in brown) of area (A) submitted to a tangential force (F) slides on the surface of the blood stream of depth (D). The shift at the surface of the blood stream (X) generates, a movement of the fluid (x). According to the formula, shear rate and SS are the key factors in determining the viscosity of blood.

The concepts of shear stress and shear rate, which are related to the flow of the bloodstream in a blood vessel, and the key parameter in the case of blood that does not meet the criteria of a Newtonian fluid—viscosity (µ)—are summarized schematically in Figure 1.

## 3. Shear Stress Sensing

Invasion/migration of enEVTs into the uterine spiral arteries persists until approximately 10–12 weeks of gestation [25]. Arterial remodeling is associated with partial narrowing or even temporary clogging of the lumen by enEVTs following the direction of blood flow, and the exponent of significant hemodynamic changes is local shear stress that changes dynamically over a wide range [30,49]. Such trophoblast plugs impede blood flow and are the main cause of the creation of a low-oxygen environment, facilitating blood vessel formation within the placental tissue through angiogenesis and vasculogenesis [50,51,52]. It has been established that the oxygen tension, or partial pressure of oxygen (PaO_2_), experienced by the trophoblast in the intervillous space in the 8th week of pregnancy is on average 17.9 mmHg (range: 5–30 mmHg), whereas at the decidua/endometrium level, it is clearly higher, averaging 39.6 mmHg (range: 25–70 mmHg) [53]. Different levels of shear stress provide many stimuli to the mechanoreceptors of endothelial cells and enEVTs that adopt a pseudoendothelial phenotype [26,30,54,55]. Understanding the relationship between the mechanosensing of shear stress by endothelial cells and trophoblasts is crucial for determining the mechanisms regulating the invasiveness of enEVTs and the remodeling of spiral arteries [20,56,57,58,59].

### 3.1. Mechanoreceptors of the Vascular Lining

Mechanoreceptors are specialized protein structures located on the surface of cells that form the lining of blood vessels and directly interact with the blood stream, the energy of which leads to the deformation of the vessel wall [56]. In this way, mechanoreceptors can receive mechanical stimuli associated with shear stress and stretch and then transform them into biochemical signals that, in response to changes in blood flow and pressure, play a key role in maintaining vascular homeostasis [60,61]. Understandably, detailed studies of mechanoreceptors focus mainly on endothelial cells and neural sensory cells (e.g., carotid sinus baroreceptors), whereas the identification of a possibly variable set of mechanoreceptors on the surface of enEVTs adopting a pseudoendothelial phenotype is more difficult and incomplete [26,62].

The previously mentioned shear stress values, which fluctuate within a wide range, and the much stronger impact of shear stress on the lining of arteries than on that of veins make the precise perception of these stimuli require differentiated mechanosensing through the participation of different types of mechanoreceptors [63,64,65,66]. The ability of endothelial cells to sense mechanical forces and mechanotransduction depends on the presence of many receptor systems, within which the functions of mechanoreceptors can be performed by

- Ion channels directly activated by mechanical stimuli;
- Structures linked to the cell membrane;
- Cytoskeletal elements;
- Junctional proteins;
- Other proteins.

#### 3.1.1. Mechanosensitive Ion Channels (MSICs)

MSICs can be defined as proteins that can convert mechanical forces within the cell into electrical or chemical signals [67]. The activators of these channels are physical stimuli such as shear stress, but also stretching and pressure, leading to the flow of ions across the cell membrane and triggering downstream signaling pathways [68]. The presence of this broad group of proteins that sense mechanical extracellular and intracellular changes, translating them into ion influx, in each cell explains the fact that all cells in an organism are mechanosensitive [69]. Moreover, cells respond not only to external mechanical stimuli but also to the forces acting within them, such as osmotic pressure and membrane deformation.

The significant and constantly increasing number of new ion channels that have been assigned mechanosensory properties has created the need to develop precise criteria to ensure that a given channel can be classified as a direct (true) mechanoreceptor (MSIC) and not as an indirect mechanosensor. The six criteria listed below, the effectiveness of which is still under discussion, were presented by two independent research teams [70,71].

- The structure of the channel includes the incorporation of a pore-forming subunit for rapid ion conduction.
- It is assumed that an isolated (purified) channel placed in an artificial, cell-free lipid bilayer will open in response to tension exerted on the bilayer.
- Directed mutagenesis within key domains of a given channel, affecting pore selectivity or conductivity, should modify mechanosensitivity.
- Enforcing the expression of a given channel in a nonmechanosensory cell is expected to result in mechanosensitivity.
- Expression of both the gene and protein of a given ion channel in a potentially mechanosensitive cell should be confirmed.
- Elimination (knockout) of a specific gene encoding the protein of a given channel makes it possible to establish that the channel not only functions in developmental processes but is also not a downstream signaling partner of another mechanosensor. The use of dominant-negative suppression of a given channel with a mutated ion channel subunit might be an improved option, considering that genetic deletion can perturb the formation of normal signaling complexes.

Fulfilling the above criteria as much as possible, several different families of mechanoreceptor channels have been established in mammals, of which the most important or best known MSICs in the endothelium include the following [67]:

- The epithelial sodium channel/degenerin (ENaC/DEG) family;
- Transient receptor potential (TRP) channels;
- Two-pore domain potassium (K2P) channels;
- PIEZO channels.

**Figure 2:**
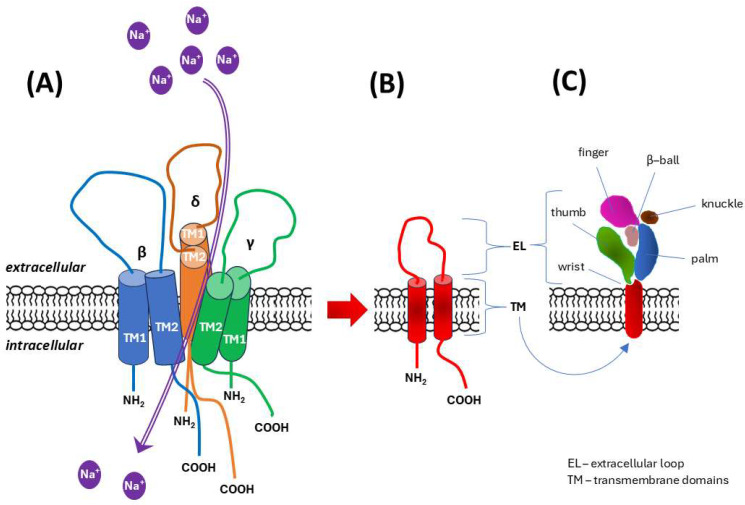
Diagram of the structure of mechanosensory channels from the epithelial sodium channel/degenerin (ENaC/DEG) family. Based on literature data [74,75,76,77]. (**A**) The channel exists as a heterotrimer, which in human endothelial cells is formed by δβγ subunits. (**B**) Overall architecture of the ENaC/degenerin subunit: two transmembrane domains (TM1, TM2) are accompanied by a large extracellular domain, and both the amino-terminus (NH_2_) and the carboxy-terminus (COOH) are located on the intracellular (cytoplasmic) side. TM2 oriented in the heterotrimer molecule towards the channel lumen plays a key role in ion selectivity for sodium (Na^+^) and channel gating. (**C**) The spatial organization of the extended extracellular domain/loop resembles the shape of an outstretched hand holding a ball, which is reflected in the names of the distinguished subdomains involved in gating and mechanosensing.

**ENaC/DEG channels.** The epithelial sodium channel/degenerin (ENaC/DEG) family of ion channels, which are known primarily for their role in epithelial cells, are also present in and potentially function in endothelial cells. ENaC/DEG channels are thought to typically be composed of αβγ or δβγ subunits, encoded by the genes *SCNN1A* (α subunit), *SCNN1B* (β subunit), *SCNN1G* (γ subunit), and *SCNN1D* (δ subunit) [72]. In the human genome, *SCNN1A* is located on the short arm of chromosome 12 (12p13.31), *SCNN1B* and *SCNN1G* are located side by side on the short arm of chromosome 16 (16p12.2), and *SCNN1D* is located on the short arm of chromosome 1 (1p36.33) [72,73]. ENaC/DEG channels are composed of trimer-forming subunits, each of which contains two transmembrane helices and a large extracellular domain rich in disulfide bonds. Structural analysis of an acid-sensing ion channel (ASIC), a member of the ENaC/DEG family, revealed that the extracellular domain contains several subdomains, the spatial arrangement of which resembles an outstretched hand holding a ball. Hence, these subdomains involved in gating and mechanosensing extracellular stimuli have names such as “palm”, “thumb” or “finger” [74]. Transmembrane domains constitute the ion permeation pathway, with the second transmembrane helix (TM2) playing a key role in ion selectivity and channel gating (Figure 2) [75,76,77].

The pores between these subunits allow Na^+^ ions to flow through the cell membrane. ENaC/DEG channels are constitutively active, voltage-independent and sodium-selective channels that can be modulated by Ca^2+^ ions. In endothelial cells, they may contribute to regulating vascular tone and permeability, influencing blood flow and fluid exchange between the bloodstream and surrounding tissues. Shear stress or stretching of the vascular wall leads to changes in cell stiffness, and the activation (opening) of mechanosensory ENaC/DEG channels is the very first step in cellular mechanosignaling [66,78].

It has been established that the expression of ENaC/DEG variants with the δ subunit in human vasculature is associated with vascular wall properties and blood pressure [79,80]. At the level of endothelial cells, ENaC/DEG channels influence vascular tone by increasing the intracellular influx of Na^+^, stabilizing f-actin (a key component of the cytoskeleton) and inhibiting endothelial nitric oxide synthase (eNOS). The stiffness of endothelial cells increases, and the production of nitric oxide (NO) is reduced, especially since the increase in the intracellular concentration of Na^+^ ions hinders the transport of L-arginine, a substrate for eNOS [81]. The involvement of selective mechanosensory sodium channel disorders in the pathomechanism of hypertension, especially salt-sensitive forms, is still under investigation because ENaC/DEG channels plays a crucial role in regulating sodium and water balance in the kidneys [81,82,83].

The vascular endothelium is the site of action of angiotensin II and aldosterone, the latter of which regulates ENaC/DEG channel expression in a mineralocorticoid-dependent manner [80].

**TRP channels.** Mechanosensitive members of this family are nonselective cation channels that play important roles in Ca^2+^ signaling [84]. In response to deformation and/or shear stress, depolarization occurs through the influx of Ca^2+^ ions. By changing the calcium level in endothelial cells, TRP channels can influence vascular tone, and subsequent dilation or constriction occurs as a result of the activation of other ion channels and signaling pathways that directly regulate vascular tone through cooperation with vascular smooth muscle cells [85]. An increase in intracellular Ca^2+^ is among the earliest events in response to shear stress in the endothelium, and the endoplasmic reticulum (ER), which acts as a storage and release site for Ca^2+^, plays a crucial role in intracellular calcium signaling via TRP, particularly in response to mechanical stimuli [68,69].

Excess Ca^2+^ activates endothelial nitric oxide synthase (eNOS) and intermediate conductance Ca^2+^-activated K^+^ channels (IK_Ca_). Vasodilation occurs mainly because of the release of nitric oxide (NO), which diffuses into vascular smooth muscle cells and activates the enzyme soluble guanylate cyclase (sGC) [86,87]. This activation leads to the production of cyclic guanosine monophosphate (cGMP), which then triggers a cascade of events that ultimately cause smooth muscle cell relaxation [88]. Moreover, by converting diverse stimuli, not only mechanical but also thermal, related to oxidative stress and the action of vasoactive factors, into changes in intracellular calcium levels that trigger downstream vascular responses, TRP channels are involved in regulating vascular tone, fluid volume, permeability, angiogenesis and endothelial secretion and proliferation [89,90,91,92].

TRP channels exist as tetrameric complexes composed of different or identical subunits, each of which has six domains with both the N- and C-termini in the intracellular compartment [93,94]. TRP expression has been demonstrated primarily in the cell membrane and ER and, to a lesser extent, in intracellular membranes, including membranes of the endolysosomal system.

In humans, 27 different TRP genes are grouped into six different subfamilies based on their amino acid homologies [95,96]:

– TRPC (canonical);
– TRPM (melastatin);
– TRPV (vanilloid);
– TRPA (ankyrin);
– TRPP (polycystin);
– TRPML (mucolipin).

The general characteristics of the TRP subfamilies and members are presented in Table 1.

Among the abovementioned TRP subfamilies, studies on mechanosensory properties have most often focused on TRPV1–TRPV6 as representatives of transient receptor potential vanilloid-type cationic (TRPV) channels, with TRPV4 likely being most studied with respect to its mechanosensing and related cell migration [170,202,203,204,205,206,207].

**Figure 3:**
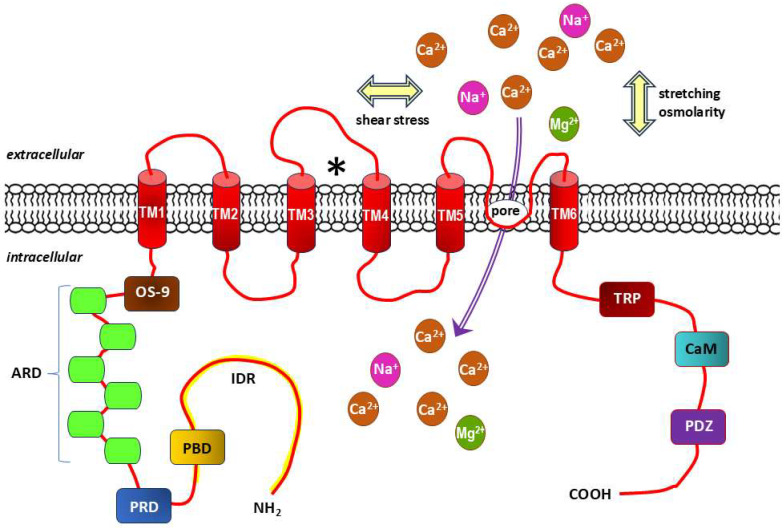
Structure diagram of transient receptor potential channel subfamily V (vanilloid) member 4 (TRPV4). Based on literature data [208,209]. Each TRPV4 subunit, which exists as a tetramer, contains 6 transmembrane domains (TM1-6), within which there is a pore-forming loop between the TM5 and TM6 domains and typical receptor agonist binding site with amino acids of the TM3 and TM4 domains (marked with “⁎”). Mechanical stimuli acting on the cell membrane related to shear stress, stretching and osmolarity also activate this non-selective cationic mechanoreceptor channel. The amino-terminus contains a lectin protein (OS-9) binding domain, six ankyrin repeats domains (ARD), a proline-rich domain (PRD) and the intrinsically disorder region (IDR, marked in yellow), while the carboxy-terminus of the protein consists of a TRP-box that stabilizes the closed state of the TRPV4 channel, a Ca^2+^/calmodulin (CaM) binding site and a PDZ-like binding domain (also known as PSD95/Dlg/ZO-1-like). Ca^2+^, Na^+^, Mg^2+^: calcium, sodium and magnesium cations, respectively.

TRPV4 channels have functionally diverse domains, including a proline-rich domain (PRD), six ankyrin repeat domains (ARD) and a lectin protein (OS-9)-binding domain in the amino terminus (NH_2_), and are stabilized in the closed state of the TRPV4 channel TRP-box, Ca^2+^/calmodulin (CaM)-binding site and PDZ-like-binding domain (also known as PSD95/Dlg/ZO-1-like) in the carboxy terminus (COOH) of the protein. Channel agonists mostly bind to the amino acids of transmembrane segments 3 and 4 (TM3 and TM4, respectively), and the channel pore is located between TM5 and TM6 (Figure 3) [208,209].

Definitive assignment of mechanical sensitivity to the above TRP subfamilies encounters significant difficulties [210,211]. Despite numerous studies linking various TRP channels to mechanosensing, evidence supporting their functions as primary transducers of mechanical forces is limited and often of questionable reproducibility [212,213]. However, it is suggested that TRP channels may have as yet undefined structural properties, predisposing them to the reception of mechanical stimuli, or act as mechanoamplifiers that respond downstream of the activation of a primary mechanotransducer, which may include Ca^2+^-permeable mechanosensitive channels or other unidentified mechanical sensors [214]. The response of TRPV1 to stretch and osmotic stimuli is also dependent on the actin cytoskeleton [215,216].

**K2P channels.** The two-pore (P1 and P2) domain K^+^ channels, also known as leak channels, have four transmembrane segments and occur as dimers of pore-forming subunits, being encoded by 15 genes in the human genome, including 3 noncoding pseudogenes [217]. Unlike voltage-gated K^+^ (K_v_) channels, K2P channels are generally considered to be voltage insensitive or resistant, which, despite some controversy, has been demonstrated by observing their resistance or reduced sensitivity to classical potassium channel blockers such as tetraethylammonium (TEA), 4-aminopyridine (4-AP), cesium (Cs^+^) and barium (Ba^2+^) [217,218]. Thus, K2P channels are potassium-gated potassium channels whose potassium efflux increases the probability that the channel will open at depolarized potentials [218].

After TWIK1, the prototypical member of the K2P family, was cloned on the basis of sequence homology to its protein, 14 subunits were subsequently cloned that showed essentially the same organization. Thus, based on structural and functional properties, six subfamilies were identified that collectively represent the 15 known members of the K2P family in humans [217,218]. These are:

– TWIK channels (two-pore domain in a weak inward rectifying K^+^ channels);
– TREK/TRAAK channels (TWIK-related K^+^ channels/TWIK-related arachidonic acid-activated K+ channels);
– TASK channels (TWIK-related acid-sensitive K^+^ channels);
– TALK channels (TWIK-related alkaline-activated K^+^ channels);
– THIK channels (TWIK-related halothane-inhibited K^+^ channels);
– TRESK channels (TWIK-related spinal cord K^+^ channels).

**Figure 4:**
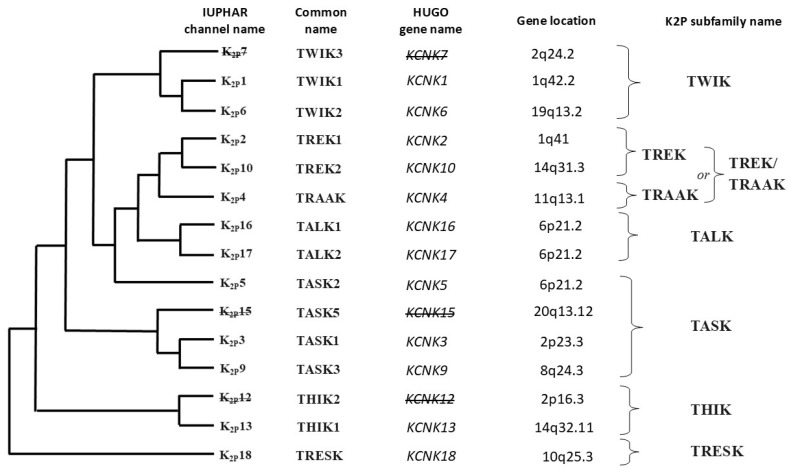
Gene tree and corresponding protein tree within the two-pore domain potassium (K2P) channel family, including subfamilies. Based on literature data [217,218]. The names of the channels are given according to the International Union of Basic and Clinical Pharmacology (IUPHAR) nomenclature. Gene names are given according to the Human Genome Organization (HUGO) nomenclature. Of the 15 genes encoding the structural subunits of K2P dimers in humans, 12 encode the corresponding proteins, while the remaining 3 genes constitute nonfunctional (noncoding) segments of DNA. The names of these pseudogenes, inactive in the human genome, and the corresponding unsynthesized K2P proteins are crossed out in the figure. TWIK—two-pore domain in a weak inward rectifying K^+^ channel, TREK/TRAAK—TWIK-related K^+^ channels/TWIK-related arachidonic acid activated K^+^ channel, TALK—TWIK-related alkaline activated K^+^ channel, TASK—TWIK-related acid-sensitive K^+^ channels, THIK—TWIK-related halothane inhibited K^+^ channel, TRESK—TWIK-related spinal cord K^+^ channel.

The phylogenetic relationships and general characteristics of the 15 K2P subunit-related genes in humans, including the nomenclature of channels within the individual K2P subfamilies, are presented in Figure 4.

Although electrophysiological recordings have shown that voltage-dependent K^+^ (Kv) channels also exhibit precise sensitivity to small (physiologically relevant in magnitude) mechanical perturbations of the cell membrane, K2P channels from the TREK/TRAAK subfamily are responsible for endothelial cells sensing shear stress and stretching of the vascular wall [219,220,221,222,223].

**Figure 5:**
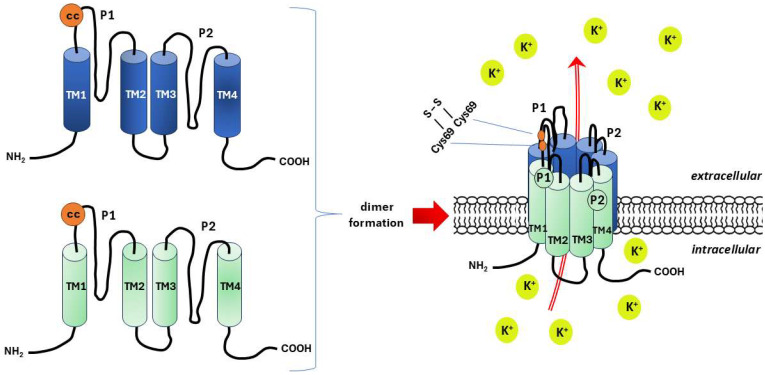
Schematic structure of the two-pore (P1 and P2) domain K^+^ channels (K2P), also known as leak channels. Based on literature data [224]. K2P forms a homo- or, less frequently, heterodimer composed of subunits with four transmembrane domains (TM1–4) and two pore domains. Coiled-coiled (cc) domain in the form of extracellular loop between the TM1 and P1 is a site of a covalent bridge (S-S) formation between the cysteine residues of both subunits (Cys69) that stabilizes the molecule of the dimer. Potassium ions (K^+^) flow in spaces bounded by helical loops of TM1P1.

As in other members of the K2P family, the N-terminus and C-terminus are located intracellularly (in the cytoplasm), and in the formed homodimer (much less frequently a heterodimer), each P domain is flanked by two transmembrane domains (TM1 and TM4). There is an extracellular loop between the TM1 and P1 domains, called a coiled-coiled domain, which serves to interact with the second subunit of the dimer with the same structure to form a covalent bridge between the cysteine residues of both subunits (Cys69) [217]. In the space between the two highly structured helical loops of TM1P1, a path is created for K^+^ ions to flow through its side branches (portals) (Figure 5) [224].

Among TREK channels, TREK-1 is distinguished; its chromosomal location is 1q41L, and that of TREK-2 is 14q31 [225,226]. Despite the 78% similarity between TREK-1 and TREK-2, there are significant differences in sensitivity to extracellular acidification and interaction with the neuropeptide spadin. TREK-1 is inhibited by extracellular acidification, whereas TREK-2 is activated. TREK-1 is also inhibited by spadin, while TREK-2 is insensitive to it [225,226].

Increased membrane tension, resulting from shear stress or cellular swelling, can activate mechanosensitivity or gating through changes in membrane tension TREK/TRAAK channels. Upon activation of these channels, an outward K^+^ current occurs, which, especially when accompanied by an intracellular influx of chloride anions (Cl^−^), may cause hyperpolarization of the endothelial cell membrane [227,228].

TRAAK is the most sensitive to mechanical stimuli, TREK-1 shows intermediate sensitivity and TREK-2 is the least sensitive. TRAAK and TREK-1 activation occurs over a wide range of stimulation intensities that encompass virtually all physiologically relevant tensions. This is in contrast to TREK-2, where the range of stimulation strengths that trigger activation is much narrower, resembling that of other mechanically sensitive channels such as PIEZO1 or bacterial small and large conductance mechanosensitive channels (MscS and MscL, respectively) [226,227,228].

**PIEZO channels.** In 2010, two piezo proteins, PIEZO1 and PIEZO2, were discovered; these proteins form large homomultimetric complexes of mechanosensitive cation channels located in the cell membrane and play key roles in the cellular mechanotransduction of mechanical stimuli into electromechanical signals [229]. When mechanically activated, these nonselective channels conduct mainly Ca^2+^ ions but can also conduct K^+^ and Na^+^ ions. Mechanical forces trigger the response of PIEZO channels within milliseconds of acting on the cell membrane, which is a significant difference compared to TRP channels, which are activated in up to 30 s [230,231,232].

**Figure 6:**
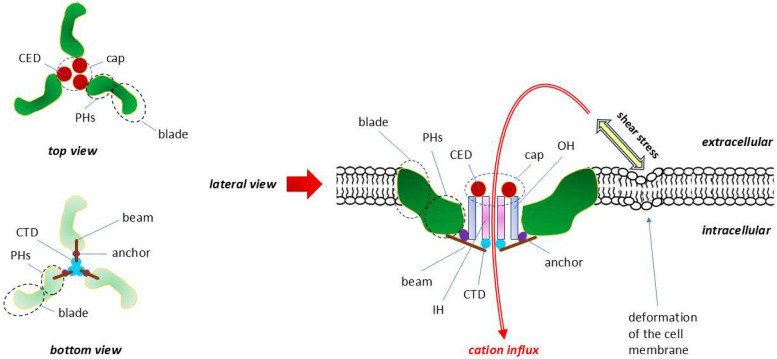
Structure and topography of the PIEZO1 receptor. Modified, based on [234,235,236]. When viewed from both the top and bottom, the subunits of this homotrimer resemble in shape and arrangement a three-bladed propeller. The central pore of the channel is surrounded by the following triples: C-terminal extracellular domain (CED), transmembrane inner helix (IH), outer helix (OH) and intracellular C-terminal domain (CTD). The spatially extended connections between the three CEDs form an extracellular cap. The intracellular beam connects the peripheral helices (PHs) to the CTD. Increased tension and/or deformation of the cell membrane resulting from mechanical forces associated with shear stress directly activate PIEZO channels and the cell. Mechanical forces are determinant of intracellular Ca^2+^ signaling. The lack of selectivity of PIEZO channels means that, in addition to the quantitatively dominant Ca^2+^ ions, they also allow other cations to pass through, such as K^+^, Na^+^ and Mg^2+^. CED—C-terminal extracellular domain, IH—inner helix, OH—outer helix.

PIEZO1 and the related PIEZO2 are large, evolutionarily conserved proteins comprising are 2521 and 2752 amino acids in humans, respectively. In humans, the Fam38A gene encoding PIEZO1 is located on chromosome 16, and the Fam38B gene encoding PIEZO2 is located on chromosome 18 [229]. Both PIEZO1 and PIEZO2 are homotrimers whose arrangement resembles a three-bladed propeller that comprises 114 transmembrane helices (38 per protomer) (Figure 6) [233].

Comparative analysis of the structures of PIEZO1 and PIEZO2 by cryo-electron microscopy revealed that the transmembrane central pore of PIEZO1 is dilated, whereas the analogous central pore of PIEZO2 is closed [233].

PIEZO1 is involved in mechanotransduction in a variety of cells, including endothelial cells and smooth muscle cells, whereas PIEZO2 plays a key role in sensing slight touch, proprioception, tactile pain, breathing and blood pressure almost exclusively via neurons (e.g., trigeminal sensory neurons, dorsal root ganglia, somatic neuron cells and epithelial neuroendocrine cells, including Merkel cells) [234]. In vascular endothelial cells, PIEZO1 is an important shear stress sensor and is involved in cell alignment in the blood flow direction [237]. Moreover, endothelial cells can recognize PIEZO1 and respond differently to the way blood flows in a vessel. In a mechanism not yet explained, they convert the mechanical signal related to laminar flow into an atheroprotective response with eNOS activation, whereas turbulent blood flow may induce a response involving NF-κB activation and inflammatory signaling [238]. The detailed mechanisms enabling the delivery of mechanical stimuli affecting the activation, inactivation and localization of PIEZO1 are under investigation. It is assumed that the forces resulting from shear stress can be transmitted as deformations of the cell membrane through lipid compounds (force from lipids) and with the participation of the entire cell cytoskeleton and extracellular matrix (ECM) components (force from filaments) [239].

The typical laminar flow-related mode of PIEZO1 activation involves the release of ATP from endothelial cells and subsequent activation of the G_q_/G_11_-coupled purinergic P2Y_2_ receptor, a member of the family of G protein-coupled receptors (GPCRs) [240,241]. Activation of downstream signaling pathways related to P2Y_2_ and G_q_/G_11_ cascades leads to flow-induced vasodilation mediated by protein kinase B (Akt) and eNOS/NO [238]. NO release and vasorelaxation were also demonstrated with Yoda 1, which is the first agonist developed for the mechanosensitive ion channel PIEZO1 [242]. It has been suggested that the PIEZO1-soluble adenylyl cyclase (sAC)-inositol 1,4,5-triphosphate receptor type 2 (IP3R2) mechanotransduction circuit plays a key role in the activation of Akt signaling as an important mechanism of the adaptive morphological response of endothelial cells to shear stress, leading to vascular dilation [243]. This is because ER Ca^2+^ release is required for the Akt-dependent response of endothelial cells to shear stress and because mechanosensing via PIEZO1 is linked to cAMP-dependent ER Ca^2+^ release through IP3R2 [243].

#### 3.1.2. Mechanosensitive G-Protein Coupled Receptors (GPCRs)

**Figure 7:**
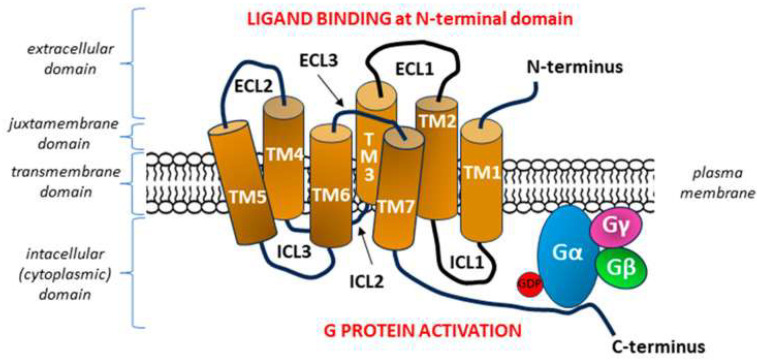
General diagram of the structure of a G protein-coupled receptor (GPCR). Adopted from [247]. The seven transmembrane heptahelical structures (TM1–TM7) are accompanied by three extracellular loops (ECL1–ECL3) on the N-terminal side of the molecule and three intracellular loops (ICL1–ICL3) on the side of C-terminal tail. After binding the signal molecule (ligand) at the N-terminal domain, conformation changes occur in the transmembrane GPCR molecule, which enable ICLs to interact with the G protein within the C-terminus located in the intracellular domain, with subsequent activation of the G protein [248]. Each G protein is composed of three subunits α, β, and γ with a nucleotide-binding pocket located in the α subunit. In the inactive heterotrimeric state, guanosine diphosphate (GDP) is bound to the Gα subunit. The formation of the GPCR—G protein complex after GPCR stimulation begins with the release of GDP from its binding site on the G alpha subunit, which is equivalent to the activation of the G protein, as it allows the binding of guanosine triphosphate (GTP) and inducing the dissociation of the α subunit of the G protein from the βγ subunits [249,250].

The expression of GPCRs was first demonstrated by Robert J. Lefkowitz in the 1970s, and research on the detailed structure and functions of GPCRs was initiated by his colleague Brian K. Kobilka. Both outstanding molecular biologists received the Nobel Prize in Chemistry in 2012 for research “crucial for understanding how G protein-coupled receptors function” [244]. The characteristic seven transmembrane (7TM) structure of a GPCR with three extracellular loops (ECL1–ECL3) and three intracellular loops (ICL1–ICL3) defines the detection of bound molecular ligands on the N-terminal outer surface of the cell to activate intracellular responses through interaction with G proteins on the C-terminal side of the receptor [245,246] (Figure 7).

The first identified member of the GPCR family with mechanosensory properties was the angiotensin II type 1 receptor (AT1R). Mechanical forces associated with, e.g., shear stress or vessel stretching, directly activate AT1R, independent of angiotensin II [251,252]. On the basis of molecular mechanics, it was established that a mechanical stimulus causes anticlockwise rotation of the transmembrane segment 7 (TM7) of AT1R into the ligand-binding pocket, indicating that TM7 is a key structural determinant of mechanosensitivity [253].

It soon became apparent that many other types of GPCRs from the Gq/11 (Gq/G11) family are capable of sensing shear stress or responding to this mechanical stimulus in a ligand-independent manner [254,255,256]. Although most of the data obtained in vitro have not been verified in vivo, putative mechanosensitive GPCRs, without distinguishing between direct mechanosensing and transduction of downstream mechanosensor signaling, include, among others, GPR68 receptor (also known as OGR1), histamine H1 receptor (H1R), endothelin B receptor (ETB), muscarinic acetylcholine receptor M5, vasopressin V1a receptor (AVPR1A), bradykinin receptor B2 (B2R), sphingosine-1-phosphate receptor 1 (S1PR1) and dopamine receptor D5 (also known as D1BR) [257,258,259,260,261,262]. In the case of all the above receptors, activation could occur because of a change in the 3D conformation of the molecule/molecules after a mechanical stimulus. Subsequent studies have shown that the key structural motif responsible for the mechanosensitivity of most GPCRs is the C-terminal helix 8 (H8), a short α-helix found in most receptors located immediately after TM7 [263].

Activation of mechanosensitive GPCRs stimulates the Gαq/11 protein, which then activates phospholipase C (PLC). PLC cleaves a membrane phospholipid, generating inositol trisphosphate (IP3), which binds to receptors on the endoplasmic reticulum (ER), causing the release of calcium into the cytoplasm. Therefore, shear stress can increase the intracellular Ca^2+^ concentration in ECs, resulting in the Ca^2+^/calmodulin-dependent activation of eNOS and the release of NO, ultimately leading to vasodilation through VSMC relaxation [254,256,264].

Recently, research on mechanosensitivity and mechanotransduction has focused on the adhesion class of GPCRs (aGPCRs), which is the second largest family of GPCRs, with 33 members in humans [265]. A characteristic feature of the aGPCR structure is the presence of a large extracellular N-region (ECR) containing variable tandem adhesion domains, followed by a common GPCR autoproteolysis-inducing (GAIN) domain. Owing to the GPCR-containing proteolytic site (GPS), a link is created with the C-terminal 7TM domain [266]. Autoproteolytic cleavage of GPS results in the formation of a bipartite structure containing an N-terminal fragment (NTF) and a C-terminal fragment (CTF), maintained by noncovalent interactions. The GAIN domain also contains a tethered agonist element (TA, Stachel peptide) at the N-terminus of the CTF. It has been shown that the dissociation of the NTF/CTF complex with TA release occurs during aGPCR activation by a mechanical stimulus. Single-molecule studies revealed that the unfolding of the GAIN domain and the release of TA were directly observed under the action of forces within the physiological range [267]. This confirms the possibility that some aGPCRs function as metabotropic mechanosensors, in which the GAIN domain may serve as a molecular integrator of mechanical forces through the dissociation of the NTF/CTF complex and the release of TA with the accompanying unfolding [266,268].

Analysis of the participation of both MSICs and GPCRs in the reception of mechanical stimuli at the level of the cell membrane provides a basis for treating the next stage—mechanotransduction—as a process encompassing the entire cell (cytoskeleton, nucleus) and its environment (junctional proteins, ECM including glycocalyx) (see Section 3.1.4) [269,270,271,272,273].

#### 3.1.3. Receptor Tyrosine Kinases (RTKs)

The human genome contains more than 90 tyrosine kinases (TKs), whose enzymatic activity allows the transfer of a phosphate group from ATP to the tyrosine residues on proteins [274]. Depending on the specificity of their action, these kinases can be divided into two groups: nonreceptor kinases (TKs) and receptor kinases (RTKs). RTKs are a family of membrane receptors involved in intracellular signaling cascades that regulate key processes such as cell proliferation, differentiation and survival [275]. Considering the similarities of the amino acid sequences of RTKs and the characteristic structural features of their extracellular domains, as well as the similarities of the respective ligands, this heterogeneous group is divided into 20 subfamilies (classes) [276,277]. While activation of most RTKs occurs after ligand binding to the extracellular domain and subsequent dimerization, activating the intracellular kinase domain, some RTKs can be activated alternatively by mechanical forces such as shear stress, cell–cell or cell–ECM interactions and ECM stiffness, without binding to a ligand. Mechanosensitive RTKs include, among others, four members of the epidermal growth factor receptor (EGFR) family, vascular endothelial growth factor receptors 2 and 3 (VEGFR2 and VEGFR3) and RTKs directly related to the cytoskeleton and contractile machinery (AXL and ROR2). The localization of these latter two receptors upon activation involves local contraction units, which resemble sarcomeres, where they bind to the major contractile structures, tropomyosin 2.1 (AXL), myosin IIA (AXL), and filamin (ROR2) [278]. The perception of changes in cell shape, tension and stiffness (deformation) is then associated with cell migration and adhesion. Moreover, signaling through AXL and ROR2, but activated by ligands, has recently attracted the attention of researchers in the processes of organogenesis, malignant tumor growth and metastasis [279,280,281].

**Figure 8:**
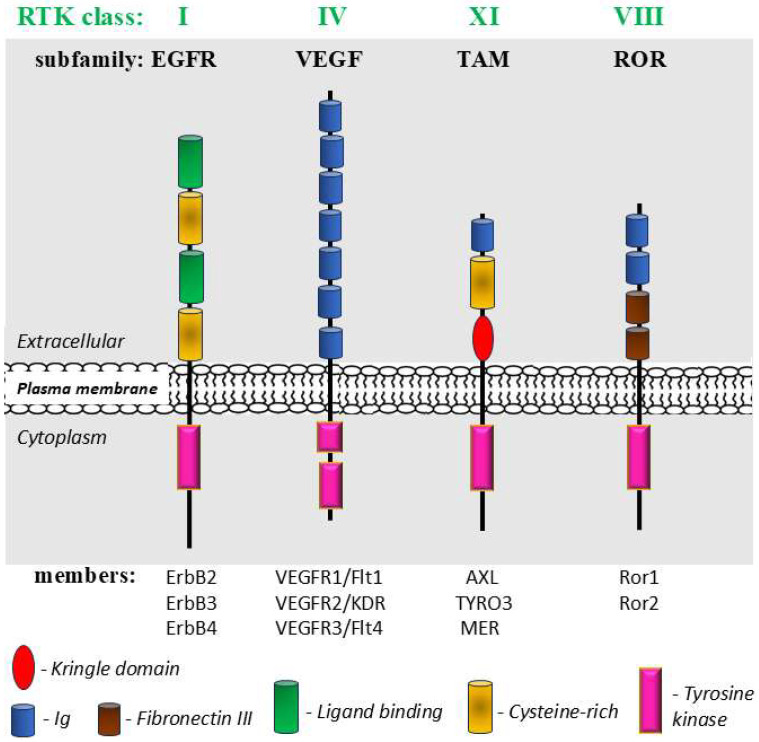
A schematic structure of well-known representatives of mechanosensitive receptor tyrosine kinases (RTKs) from 4 classes: epidermal growth factor (EGFR or ErbB) subfamily (class I); vascular endothelial growth factor (VEGF) subfamily (class IV); receptor tyrosine kinase-like orphan receptor (ROR) subfamily (class VIII) and TAM receptor subfamily (class XI). Modified, based on [277,281,282,283].

The general structure of RTKs, considering significant differences, primarily in the structure of their extracellular domains, is shown in Figure 8. Of the 20 classes of RTKs, representatives of 4 were selected, with well-documented mechanosensing and mechanotransduction [277,282].

#### 3.1.4. Mechanotransduction and Whole-Cell Mechanosensing

The perception of a mechanical stimulus by mechanoreceptors or mechanosensing requires a whole-cell response, which is achieved by mechanotransduction [269]. In the process of mechanotransduction, in which various structures, such as the plasma membrane, cytoskeleton, extracellular matrix (ECM) and intracellular organelles, participate, the received stimuli are converted into biochemical signals for a given cell and neighboring cells [66,284]. Ultimately, information from the outside in the form of a mechanical stimulus is transmitted intracellularly, influencing gene expression and cellular behavior [285,286].

Conformational changes in cell membrane mechanoreceptors under the influence of shear stress, hydrostatic pressure and/or circumferential stretch can occur directly when at least one of the mechanoreceptor ends is anchored to relatively firmly fixed intracellular or extracellular structures and when the other end is pulled in line with the force vector [287]. The stable intracellular anchor points of mechanoreceptors may be the cytoskeleton and the intracellular membranes of the organelles, including the nuclear membrane, whereas the three-dimensional network of macromolecules that form the ECM may serve as an external attachment point [288,289]. An alternative, indirect way of stimulating mechanoreceptors may be the stretching/tensioning of the surrounding cell membrane under the influence of an acting force, which leads to a change in the positioning of the receptor in the lipid bilayer [290,291].

The cellular machinery for receiving mechanical forces relies heavily on focal adhesions (FAs), which are large macromolecular assemblies that essentially connect the intracellular cytoskeleton with the ECM [292]. Conformational changes in proteins located in FA areas initiate signaling cascades crucial for mechanotransduction. The key proteins within FAs are Src protein-tyrosine kinase, integrins, talin, kindlin, vinculin, paxillin, focal adhesion kinase (FAK), and integrin-linked kinase (ILK) [293,294,295].

The cytoskeleton forms the scaffolding of the cell, which mainly determines its shape and mechanical resistance to deformation, but at the same time, it retains high plasticity and exhibits dynamic changes under the influence of mechanical stimuli [296,297]. This dynamic nature of the cytoskeleton, which forms a highly anisotropic network extending from the cell nucleus to the cell membrane and is subject to constant remodeling, results from the differential polymerization and depolymerization of the filaments that compose it, mainly actin and myosin, and, to a lesser extent, microtubules and intermediate filaments [66,298]. As a result of mechanotransduction, the cytoskeleton undergoes remodeling, which changes its composition and/or spatial arrangement (intracellular orientation) so that the cell can optimally accommodate the forces related to shear stress, tension and stiffness and respond to them appropriately [299,300]. The physiological range of changes in the mechanical properties of endothelial cells from “stiff” to “soft” is strikingly wide. Therefore, the degree of deformation of an endothelial cell or pseudoendothelial cell of the invasive trophoblast under the influence of mechanical stimuli related to blood flow depends on both the expression of mechanoreceptors in the cell membrane and the mutual proportion of receptor proteins tightly or loosely associated with the cytoskeleton [301,302].

Mechanosensing pathways can also be activated at the interface between the cell membrane and the ECM, where interactions of basic ECM components such as glycosaminoglycans (GAGs), especially heparan sulfate (HS) and chondroitin sulfate (CS), and proteoglycans (ProGs) with mechanoreceptors modulate excitability and responsiveness to mechanical stimuli [303,304]. Impaired functioning of GAG/ProG-mediated mechanosensing pathways has been demonstrated in many pathological conditions, including inflammation and cancer [56,305].

A particular form of ECM specialization is the endothelial glycocalyx, which covers the apical side of vascular endothelial cells and extends into the lumen of blood vessels, acting not only as a passive physical barrier between the vascular wall and blood but also as a dynamic multifunctional structure [306]. Heparan sulfate proteoglycans (HSPGs), glypican-1 and syndecan-1, found in the glycocalyx on the cell surface are involved in regulating cell signaling and growth factor interactions, and the positioning of these glycocalyx components relative to the major glycosaminoglycans HS and hyaluronic acid under the influence of mechanical forces (e.g., increased shear stress) is responsible for endothelial mechanosensing with subsequent mechanotransduction [306,307].

Importantly, the placental syncytiotrophoblast also has a glycocalyx in direct contact with the mother’s blood, analogous to the endothelial glycocalyx [308,309]. Considering the location of the syncytiotrophoblast, its epithelial-like nature, and its barrier function in the placenta, it can be assumed that the placental glycocalyx does not differ significantly in composition from the endothelial glycocalyx and performs similar functions, including participation in mechanosensing/mechanotransduction [306]. For example, syndecans have been shown to be present in large quantities in both glycocalyxes, and elevated serum syndecan-1 concentration is a marker of both endothelial and placental villus epithelial dysfunction and indicates degradation of their glycocalyxes [310,311,312].

**Figure 9:**
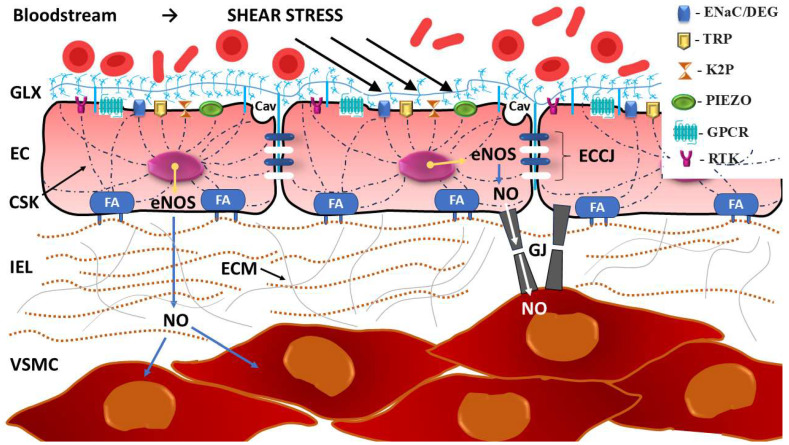
Whole-cell mechanosensing as a key phenomenon in mechanotransduction: cellular and extracellular components. Mechanosensing of stimuli related to blood flow, such as shear stress, occurs through mechanoreceptors in the form of mechanosensitive ion channels (MSICs) and G protein-coupled receptor (GPCRs). The conversion of mechanical stimuli into electrochemical or biochemical signals occurs in the transduction process involving the entire cell, including structures linked to the cell membrane, cytoskeletal elements, glycocalyx (GLX), junctional and other proteins. Focal adhesions (FA), formed by dynamic protein structures containing integrins, establish connections between the intracellular cytoskeleton (CSK) and the extracellular matrix (ECM), which favors the propagation of deformation as a result of mechanical force. Mechanotransduction also influences cell behavior and function by changing the level of protein synthesis in the cell nucleus. For example, upregulation of endothelial nitric oxide synthase (eNOS) increases the production of nitric oxide (NO), which diffuses through the cell membrane, and especially through myoendothelial gap junctions (GJ), leading to endothelium-dependent relaxation of vascular smooth muscle cells (VSMC). Cav—caveola, EC—endothelial cell, ECCJ—endothelial cell–cell junctions, ENaC/DEG—epithelial sodium channel/degenerin family of mechanosensory channels, IEL—internal elastic lamina, K2P—two-pore domain potassium channels; PIEZO—PIEZO channel, RTK—receptor tyrosine kinase, TRP—transient receptor potential channel. Considering the response to vascular shear stress as a phenomenon encompassing the entire cell and the multidirectional nature of mechanotransduction, it is not surprising that a complex network of cell signaling pathways is activated. Conformational changes in proteins within FAs, the mitogen-activated protein kinase (MAPK) pathway, the phosphorylation of extracellular signal-regulated kinases (ERKs), and the GPCR-related mechanosensing Gq/11/PLC calcium signaling pathway are involved [33,256,313]. Moreover, rapid tyrosine phosphorylation of platelet endothelial adhesion molecule-1 (PECAM-1) triggers a pathway associated with angiogenesis and maintaining the homogeneity of ECs, in which VEGFR2/VEGFR3, vascular endothelial cadherin (VE-cadherin, also known as CDH5), phosphatidylinositol 3-kinase (PI3K), and protein kinase B (PKB, also known as Akt) participate [33,314,315,316,317,318].

The cellular and extracellular components involved in the mechanotransduction process are shown in Figure 9.

The activation of diverse signaling pathways in response to mechanical stimuli indicates that the cell nucleus actively participates in mechanotransduction [319]. Moreover, owing to the physical connection of the nucleoskeleton with the cytoskeleton in the form of the nucleoskeleton and cytoskeleton (LINC) complex as a linker, mechanical forces acting on the cell surface are transferred to the nucleus. The cell nucleus is the largest and stiffest organelle [273]. Studies on isolated cell nuclei have confirmed their ability to respond to force by adjusting their stiffness to resist applied tension. Under the influence of tensioning the LINC complex component nesprin-1, tyrosine phosphorylation occurred in emerin, a protein of the inner nuclear membrane, with a subsequent mechanical response of the nucleus in the form of stiffening of its structure. This stiffening of the nucleus did not involve chromatin or nuclear actin but required intact nuclear lamina [320].

## 4. Shear Stress and Uterine Spiral Artery Remodeling by enEVT Cell Invasion

In humans, invasive trophoblasts refer to EVTs, which are derived from progenitor cells residing in extravillous cell columns. Mechanosensing studies on invasive cells, such as those responsible for the growth and spread of malignant tumors, but also on enEVTs of a similar nature, have recently provided an increasing amount of data potentially very useful in oncology and the physiology/pathophysiology of pregnancy, respectively [321,322,323,324,325].

The peculiarity of the growth of invasive enEVTs in the uterine spiral arteries is that it occurs against the blood flow [326]. This may cause a fundamental difference in the perception of mechanical stimuli during the replacement of ECs, especially those related to shear stress. However, these differences are not sufficiently understood. enEVTs migrate along, replace ECs and lead to enEVT-dependent VSMC removal and uterine immune cell death [26,327,328]. Both processes occur as a result of programmed cell death (apoptosis) after the cell surface death receptor Fas (also known as CD95 or APO1) binds to Fas ligand (FASL) and triggers the apoptotic cascade. In addition to apoptosis, which is generally considered a noninflammatory process, in vitro studies in cocultures of trophoblasts and human uterine microvascular endothelial cells (UtMVECs) have shown that under shear stress conditions, trophoblasts induce an inflammatory response in UtMVECs that can enhance trophoblast invasion and transmigration. The proinflammatory response is associated with changes in the distribution of endothelial intercellular adhesion molecule-1 (ICAM-1) and an increase in endothelial permeability [329]. Importantly, both apoptosis and the proinflammatory environment generate mechanical forces and mechanotransduction [330,331].

enEVTs exhibiting a pseudoendothelial phenotype have been shown to be mechanosensitive [322]. The mechanism by which enEVTs acquire this phenotype is unknown. It was proposed that during differentiation, a small population of trophoblast stem (TS) cells transdifferentiate into a hybrid cell type expressing markers of both trophoblasts and ECs [332]. The same study revealed that differentiating trophoblasts secrete tumor necrosis factor-related apoptosis-inducing ligand (TRAIL/Apo2L), whereas its death receptor DR4 is expressed only in ECs. Thus, TRAIL may induce apoptosis in ECs but not in enEVTs [332]. One study even questioned the endothelial mimicry of enEVTs, with the authors postulating that ECs are only transiently lost from remodeling vessels [49].

enEVTs, which are crucial for ensuring low-resistance blood flow at the implantation site, express various receptors, including MSICs, GPCRs, and tyrosine kinase mechanoreceptors (RTKs), as well as integrins and other specialized proteins for sensing mechanical stimuli from the ECM (glycocalyx) [322,323,333]. These receptors are important for ensuring interactions with the maternal environment at the site of implantation; in particular, they regulate the invasiveness of enEVTs and their migration [334,335]. However, detailed comparative data concerning mechanotransduction pathways and the resulting properties of enEVTs, e.g., migration in the direction opposite to the blood stream, remain at the research stage [331,336].

### 4.1. PIEZO1 Signaling in Trophoblast Fusion and Spiral Artery Remodeling

Both human fetoplacental ECs and enEVTs express PIEZO1 channels [322,337]. Immunofluorescence analysis revealed PIEZO1 expression in human chorionic villi, both in single-nucleated cytotrophoblasts (CTBs) and in multinucleated syncytiotrophoblasts (STBs) [322]. Furthermore, quantitative reverse transcription (qRT)-PCR analysis confirmed the expression of *PIEZO1* and *PIEZO2* mRNAs in human trophoblast stem cells (hTSCs) and BeWo cells, human placental cells originating from a chorionic carcinoma that are widely used as an in vitro model for the placenta [322]. The above results are further supported by the Comparative Transcriptome Placental Model Atlas, which lists the distinct expression of *PIEZO1* transcripts in human primary trophoblasts and hTSCs and in various human trophoblast models in vitro, such as the BeWo, JAR, JEG3, and HTR cell lines [338].

Knowledge about the role of PIEZO1 in mechanobiology and developmental processes is constantly expanding [339,340,341]. Evidence of PIEZO1 activity in trophoblasts was obtained by recording mechanosensitive current from BeWo cells with a unitary typical conductance of 29.3 ± 1.8 picosiemens (pS) using pressure clamp electrophysiology [322,342]. Consequently, when the PIEZO1 inhibitor GsMTx4 is used, intracellular Ca^2+^ influx [(Ca^2+^)_i_] can be effectively limited; this also occurs after the administration of the PIEZO1 agonist Yoda1. Both the mechanosensitive current and the increase in [(Ca^2+^)_i_] induced by Yoda1 disappear after *PIEZO1* silencing in BeWo cells and hTSCs with the appropriate siRNA [322].

Confirmation of the functional expression of PIEZO1 in human trophoblast cells significantly expands the possibility of interpreting the importance of mechanosensing under conditions of postimplantation vascular remodeling within spiral arteries [337].

PIEZO1 deficiency in *PIEZO1* knockout (KO) mice manifests as endothelial dysfunction and defective angiogenesis, leading to lethal complications at the embryonic stage [28,337]. Embryonic death in mice with constitutive KO of *PIEZO1* in trophoblasts begins as early as day 11–12 of embryonic development. Unlike the severe angiogenic defects observed in constitutive PIEZO1 KO placentae and in endothelial-specific constitutive PIEZO1 KO placentae, the trophoblast-specific deletion of PIEZO1 does not significantly affect fetoplacental blood vessel development. Unlike angiogenic disorders, defective trophoblast differentiation is the main cause of embryonic lethality in trophoblast-specific *PIEZO1* KO mice [322]. In vitro studies have shown that PIEZO1 signaling is essential for trophoblast fusion (syncytialization) in vitro, a key step during trophoblast differentiation, the disruption of which results in placental insufficiency, intrauterine growth retardation (IUGR), and, in the most severe cases, fetal death [343,344,345].

PIEZO1 mechanosensing leading to trophoblast fusion is mediated by the coupling of PIEZO1 to transmembrane protein 16 F (TMEM16F), a Ca^2+^-activated phospholipid scramblase (CaPLSase), which mediates the externalization of phosphodylserine (PS), a major signal that initiates cell fusion [322,346,347]. The importance of PIEZO1 and TMEM16F coexpression in trophoblasts is confirmed by the fact that deficiency of either protein in syncytiotrophoblast layer 2 cells (SynT-2 STBs), derived from trophoblast stem cells (TSCs) during early embryogenesis, leads to severe defects in Syn-2 STB layer development accompanied by embryonic or perinatal lethality [346,348]. It is likely, although not confirmed by studies, that similar to endothelial cells, in enEVTs with a pseudoendothelial phenotype, the expression of the scramblase TMEM16F regulates angiogenesis via intracellular signaling [349].

Trophoblast fusion can also be achieved by Ca^2+^ influx through the Ca^2+^-permeable mechanosensitive ion channel TRPV4, which can also be coupled to TMEM16F [350].

These findings suggest that mechanical forces can directly activate both PIEZO-TMEM16F and TRPV4-TMEM16F coupling, having a profusiogenic effect on trophoblasts, where the TMEM16F current depends on extracellular Ca^2+^ [346,350].

While mechanical stimuli of a compressive nature undoubtedly play a role in the fusion of CTBs to STBs, the direct effect of shear stress seems to be very limited [351]. This is because CTB-STB fusion occurs mainly on the basolateral side of STBs, which is deprived of contact with flowing blood [343]. Mechanical stimuli activating PIEZO1 may in this case be transmitted through fusogenic synapses, analogous to those observed in myoblast fusion [352,353]. The principles of mechanotransduction during trophoblast fusion undoubtedly require further investigation. Considering the central role of trophoblast fusion in placentation and subsequent placental function, such studies on placental mechanobiology may be helpful in determining the pathomechanism of pregnancy disorders such as fetal death, preeclampsia, or fetal growth restriction (FGR) [354,355].

In enEVTs, which differentiate from the CTB lineage and grow in an invasive manner, locally overlapping with endothelial cells, PIEZO1 responds to shear stress from the blood stream [28]. With respect to endothelial cells in the uterine circulation, mechanosensing and PIEZO1 activation lead to vasodilation via NO, which is accompanied by upregulation of PIEZO1 molecular expression during pregnancy [356]. Further research is needed to determine whether invasive enEVTs behave similarly. This may be important in understanding the contribution of mechanosensing disorders to the pathogenesis of preeclampsia. PIEZO1 alone can induce the phosphorylation of eNOS in fetoplacental ECs in vitro, whereas the combined activation of PIEZO1 and TRPV4 affects only eNOS phosphorylation in ECs isolated from early-onset preeclampsia patients [206].

Current knowledge of mechanoreceptors responsible for mechanotransduction in trophoblasts at various stages of differentiation and placental formation is limited to PIEZO1, TRPV4 and RTKs. However, owing to the transformation to the pseudoendothelial phenotype, mechanosensing in enEVTs may be mediated by a more diverse set of receptors, similar to that observed in endothelial cells [326]. The participation of PIEZO1, TRPV4 and RTKs in the differentiation of trophoblast and fetoplacental endothelial cells may be evidenced by the variable expression of these mechanoreceptors (Table 2).

### 4.2. Calveolae as Mechanosensors and Mechanotransducers During EVT Migration and Differentiation

In cells exposed to stretching, including shear stress, rosette-shaped, highly plastic, and flask-shaped invaginations of the plasma membrane called calveolae (Latin for “little caves”) are abundant (see Cav in Figure 9) [358]. Caveolae are not receptors but are a type of lipid signaling platform (raft) that organizes and clusters various receptors, including GPCRs, RTKs and steroid hormone receptors, along with their downstream signaling molecules [359]. Caveolae components can pass through mechanotransduction signaling pathways from the cell membrane to the nucleus to convey stress information [358]. Caveolae also maintain cell homeostasis when exposed to mechanical forces by orchestrating responses that modify ECM properties through both physical remodeling of the ECM, where the actin cytoskeleton is a central player, and chemical alteration of the ECM composition by exosome deposition [360]. In terms of mechanotransduction, caveolae constitute membrane nanodomains 50–100 nm in size that are critical for the organization and synchronization of different cellular processes, such as cell migration, invasion, and differentiation. Moreover, calveolae protect cells from mechanical stress-induced damage. Increased cell membrane tension causes flattening of the caveolae, which enables force sensing and accommodation [361]. Caveolae are areas rich in cholesterol and other lipids and proteins that interact in coordinated ways, allowing the formation of specific structures in the cavities of the cell membrane. The locally altered morphology of the cell membrane is due primarily to the presence of caveolin, the main integral membrane 21 kD protein within the caveolae, which is involved in receptor-independent endocytosis. Three homologous genes of caveolin are expressed in mammalian cells: *CAV1*, *CAV2* and *CAV3* [358,360]. It has been shown that knockout of *CAV1* and *CAV3* leads to complete loss of caveolar production, whereas deletion of *CAV2* does not affect caveolar formation [362,363,364]. Similar in structure, containing a cytoplasmic N-terminus with a scaffolding domain, a long hairpin-shaped transmembrane domain, and a cytoplasmic C-terminus, all three caveolins are synthesized as monomers and transported to the Golgi apparatus. During the secretion process, caveolins bind to lipid rafts and form 14–16-molecule oligomers, which then form caveolae [365].

Properly maintained caveolar function is essential to enable EVT migration, tubulogenesis and invasion. Aquaporin 3 (AQP3), a membrane transporter of water and glycerol expressed in the plasma membrane that plays a crucial role in facilitating trophoblast migration and invasion during early pregnancy through the fetal–maternal interface, requires an intact caveola in EVTs [366,367]. This is a sine qua non condition for the function of the methyltransferase-like 14 (METTL14)/insulin-like growth factor 2 mRNA-binding protein 1 (IGF2BP1)/AQP3/PI3K/AKT signaling pathway [368]. Disturbances in the interaction between caveolin-1 and AQP3 reduce the invasive ability of enEVTs and may contribute to a significant number of unexplained recurrent abortions. For example, hyperosmolarity at the implantation site has been shown to induce the internalization of caveolae into the cytoplasm and increase their turnover, which limits the normal differentiation of EVTs [369].

### 4.3. Trophoblast Plugs and Mechanosensing

During most of the first trimester of pregnancy, enEVTs form plugs in the lumen of spiral vessels, which dramatically affects hemodynamics within the spiral and radial arteries by restricting blood flow to the intervillous compartment surrounding the placental villi [25]. Trophoblast plugs generate shear stress conditions of <2.0 dyne/cm^2^, which promotes enEVT-induced arterial remodeling [25,27]. Although porous trophoblast plugs are permeable to blood plasma from approximately the 5th–6th week, owing to the pore diameter, they significantly limit the flow of oxygen-containing maternal red blood cells to the placenta [370,371]. Transient physiological hypoxia occurs, which is beneficial for early placental development, trophoblast differentiation, and local angiogenesis [372]. Angiogenesis may also result from mechanotransduction in response to shear stress due to the stimulation of RTKs, particularly VEGFR2 [373]. Increased ECM stiffness under shear stress heightens the downstream endothelial cell response to VEGF by altering VEGFR2 internalization [374]. Plugs may also play important roles in promoting trophoblast migration into spiral vessels and inducing EC and VSMC apoptosis with subsequent remodeling of these vessels. The increase in stiffness of the ECM, or glycocalyx, in the immediate vicinity of the cell membrane provides mechanical stimuli that promote invasive behaviors of trophoblasts [323].

It is important to remember that many properties of invasive trophoblasts may result from, occur concurrently with, or be modulated by shear stress mechanosensing. For example, mechanical stimuli (shear stress) may regulate endothelial cell apoptosis in the circulatory system [330,374,375].

In addition to apoptosis, which is generally considered a process without an inflammatory component, features of an inflammatory response are also observed at the site of trophoblast invasion [376,377]. Endothelial mechanotransduction signaling pathways participate in the generation of redox signals in response to local changes in blood flow (shear stress) that affect the oxidant and inflammatory status of cells [331,378].

The restrictions in intraplacental blood flow persist from weeks 6 to 12, which, in addition to stimulating angiogenesis. also serve to protect the delicate villi from mechanical forces associated with blood flow and oxidative stress and disappear quite rapidly at week 13 [55]. Physiological hypoxia and the subsequent restoration of the supply of well-oxygenated blood may, by themselves, independently of the accompanying changes in shear stress values, affect the differentiation and properties of the trophoblast [379,380].

Failure to establish a low-resistance uterine vasculature during the first trimester of pregnancy, owing to insufficient penetration of enEVTs into the spiral arteries, may limit placental blood flow and result in disorders of pregnancy, including preeclampsia and FGR [381,382]. Therefore, in the mechanobiology of human reproduction, trophoblast plugs and the associated changes in the mechanoreception of shear stress during the first trimester of pregnancy are crucial for the development of a functional vascular system of the uteroplacental-fetal unit [372].

**Figure 10:**
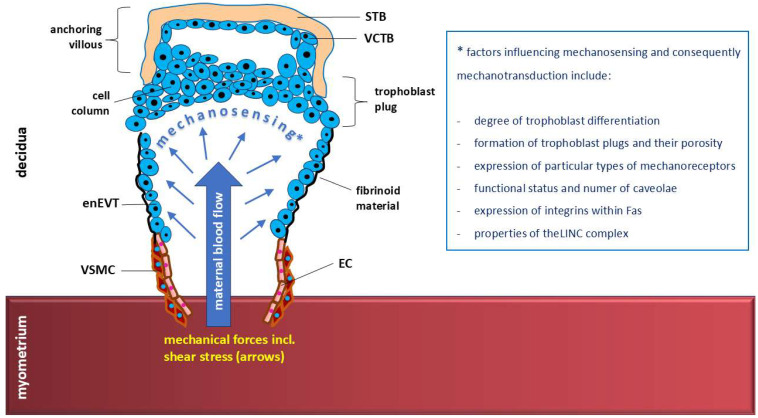
The diverse factors influencing mechanosensing of shear stress under specific hemodynamic conditions accompanying extravillous trophoblast invasion into uterine spiral arteries. The response of trophoblast cells to mechanical stimuli (mechanotransduction) may therefore be modulated to a large extent, including disturbances in trophoblast invasion. EC—endothelial cell, enEVT—endovascular extravillous trophoblast, STB—syncytiotrophoblast, VCTB—villous cytotrophoblast, VSMC—vascular smooth muscle cell.

The diverse factors influencing shear stress-related mechanosensing and mechanotransduction by EVTs are summarized in Figure 10.

## 5. Concluding Remarks

The development of low-resistance blood circulation within the uterus and placenta during early pregnancy determines its further course. The migration, differentiation and invasion of EVTs are controlled by a complex network of autocrine and paracrine factors (e.g., cytokines and growth factors), maternal decidual cells, ECM components, and, at the stage of direct contact with flowing blood during enEVT invasion into spiral arteries, mechanotransduction. Physiological trophoblast plug structures are compact enough to restrict the flow of oxygenated blood to the intervillous space during the first trimester. A local environment is then created where shear stress strongly affects the plugs, which promotes spiral artery remodeling with additional hypoxia-induced angiogenesis.

Studies on the mechanosensing of EVTs, largely based on the identification of mechanoreceptors and their associated signaling pathways, have consistently taken into account the functional similarity of EVTs to ECs in terms of angiogenesis, vascular remodeling, and cell–cell communication. Moreover, invasive enEVTs adopt a pseudoendothelial phenotype and endothelial-like functions in the placenta, a phenomenon called vasculogenic mimicry. The most advanced studies have focused on the involvement of signaling via PIEZO1 receptors in trophoblast fusion and spiral artery remodeling.

It has been shown that the possibility of sensing mechanical force (shear stress) by enEVTs is very wide and results from the stimulation of both individual receptors and whole-cell mechanosensing. To achieve optimal mechanotransduction and protection of the EVT membrane against excessive stress, caveolae are also necessary, which, although they are not mechanoreceptors, enable force sensing and accommodation.

Further research is needed on the role of shear stress in human trophoblast differentiation and placentation in the context of establishing the proper maternal–fetal blood flow necessary for a healthy pregnancy. Understanding the importance of mechanosensing disorders in trophoblasts in the pathogenesis of unexplained recurrent abortions or preeclampsia may be helpful in the development of new therapeutic strategies based on the regulation of mechanotransduction in response to shear stress.

## Figures and Tables

**Table 1:** Characteristics, distribution and main functions of individual members of the six transient receptor potential (TRP) ion channel subfamilies in humans.

TRP Subfamily	Members	Gene Location	Distribution (Cells, Tissues and Organs)	Main Functions	References
TRPC (canonical)	TRPC1	3q22–q24	Widely and evenly distributed in vascular smooth muscle cells (VSMC), heart muscle, skeletal muscles, brain (pituitary gland, cerebellar hemisphere, frontal cortex, amygdalia, hippocampus, hypothalamus, substantia nigra, anterior cingulate cortex), testis, ovary, uterus, fallopian tube, tibial nerve, liver spleen, kidney, lungs	Vasoconstriction; mechanosensing; inhibition of tumor growth and metastasis; intensification of the immune response and proinflammatory effects; myoblast migration, fusion and differentiation; regulation of cardiac plasticity and promotion of cardiac hypertrophy	Kim et al., 2019 [97]; Zhang et al., 2023 [98]; Numaga-Tomita and Nishida 2020 [99]; Elzamzamy et al., 2020 [100]; Louis et al., 2008 [101]
	TRPC2	7, 50.0 cM	In humans encoded by *TRPC2* pseudogene—the respective protein is not expressed in humans.	No function in humans (expressed in mice: involvement in the regulation of thyroid function and olfactory sensations)	Löf, C. et al., 2011 [102]; Yildirim and Birnbaumer 2007 [103]
	TRPC3	4q27	Endothelial cells, brain (mainly), heart, skeletal muscles, kidney, ovary, placenta, lungs, testis, prostate, small intestine, colon	Vasodilation; mechanosensing; regulation of vascular tone; cell growth and proliferation; wound healing and pathological hypertrophy; immune regulation	Sierra-Valdez et al., 2018 [104]; Tang, Q. et al., 2018 [105]; Patel et al., 2025 [106]; Thilo et al., 2008 [107]
	TRPC4	13q13.1–q13.2	Vascular system (endothelial cells aorta, coronary artery), brain (pituitary gland, cerebellar hemisphere, frontal cortex, amygdalia, hippocampus, hypothalamus, substantia nigra, anterior cingulate cortex), liver, adrenal gland, retina, testis, placenta, kidney, sigmoid and transverse colon	Endothelial permeability; angiogenesis; vasodilation; neurotransmitter release and cell proliferation; thermoregulation (induction of nonshivering thermogenesis)	Kim et al., 2019 [97]; Zeng et al., 2021 [108]; Cornman 2025 [109]
	TRPC5	Xq23	Brain mainly (pituitary gland, cerebellum, frontal cortex, amygdalia, hippocampus, hypothalamus, substantia nigra, anterior cingulate cortex), liver	Cell proliferation; angiogenesis; endothelium-dependent vasoconstriction; regulation of blood pressure; thermosensing; promotion of extracellular vesicle formation	Kim et al., 2019 [97]; Zeng et al., 2021 [108]; Ptakova and Vlachova 2024 [110]; Ma et al., 2014 [111]
	TRPC6	11q21–q22	Lungs, brain, myocytes, ovary, placenta	Mechanosensing; neuroprotective effects; development of glomerular injury and glomerular sclerosis; immune regulation	Tang et al., 2018 [105]; Corteling et al., 2004 [112]; Zhang et al., 2023 [98]; Staruschenko et al., 2019 [113]; ‘t Hart et al., 2023 [114]
	TRPC7	5q31.2	Heart, lungs, spleen, brain, eye, testis	Nociceptive mechanosensing; single fertilization; may contribute to heart failure as an initiator linking angiotensin receptor (AT1) activation to myocardial apoptosis	Zhang et al., 2016 [115]; Zhang et al., 2023 [98]; Hsu et al., 2020 [116]; Satoh et al., 2007 [117]
TRPM (melastatin)	TRPM1	15q13–q14	Retina (dendrites of retinal ON-bipolar cells), skin (melanosomes of melanocytes), testis (cilia of early spermatids)	An essential role in the depolarizing photoresponse of retinal ON-bipolar cells; melanin synthesis; regulation of normal and malignant melanocyte behavior; promotion of the tumor progression and malignant transformation via activating the Ca^2+^/CaMKIIδ/AKT pathway in acral melanoma; sperm development	Guo et al., 2012 [118]; Hsieh et al., 2023 [119]; Darszon et al., 2012 [120]
	TRPM2	21q22.3	Wide variety of tissues including brain, spleen, lungs bone marrow, immune cells (macrophages), heart, vasculature (endothelial cells), endocrine cells	Biosensor of reactive oxygen species (ROS), which mediates some body’s responses to oxidative stress, including maintenance of mitochondrial function, immune response, insulin secretion, body temperature control and neuronal cell death	Faouzi and Penner 2014 [121]; Xia et al., 2019 [122]; Pan et al., 2020 [123]; Cheung and Miller 2017 [124]
	TRPM3	9q21.11	Dorsal root ganglia, brain (e.g., pituitary), kidney, pancreas (the islet β-cells), eye, heart (cardiomyocytes).	The pain receptor (inflammatory pain sensation) and thermoreceptor (heat sensation) in somatosensory neurons, regulation of neurotransmitter release, maintaining glucose homeostasis through the regulation of insulin secretion by pancreatic β-cells, iris constriction, and promotion of tumor growth	Thiel et al., 2017 [125]; Vangeel L et al., 2020 [126]; Turgambayeva et al., 2023 [127]
	TRPM4	19q13.33	Wide variety of cells/tissues including prostate, small intestine and colon, heart, kidney, testis, skin, pancreas, placenta, liver, thymus, spleen	Crucial role in regulating diverse cellular functions associated with intracellular Ca^2+^ homeostasis/dynamics, including electrical activity of cardiomyocytes by depolarizing the membrane, regulation of smooth muscle contraction and immune response (e.g., regulation of Ca^2+^ oscillations after T cell activation, regulation of dendritic cell migration); promotion of tumor growth	Hu et al., 2023 [128]; Yu et al., 2024 [129]; Barbet et al., 2008 [130]; Borgström et al., 2021 [131]
	TRPM5	11p15.5	Intestine, liver, lungs, taste receptor cells, pancreas (the islet β-cells), olfactory and vomeronasal systems	Sweet, bitter and umami taste sensation (sensory transduction in taste cells); modulation of insulin secretion by pancreatic β-cells; immune response (e.g., negative regulation of LPS-induced proliferative and inflammatory response in B cells); constriction of cerebral arteries	Dutta et al., 2018 [132]; Vennekens et al., 2018 [133]; Richter et al., 2024 [134]; Sakaguchi et al., 2020 [135]; Guinamard et al., 2011 [136]
	TRPM6	9q21.13	Kidney, small intestine, colon, heart (e.g., atrial fibroblasts and cardiomyocytes)	Magnesium uptake and homeostasis in kidney and intestine; regulation of cardiac function;	Chubanov et al., 2005 [137]; Schlingmann et al., 2007 [138]; Andriulė et al., 2021 [139]; Gwanyanya et al., 2021 [140]
	TRPM7	15q21	Kidney, heart (e.g., atrial fibroblasts and cardiomyocytes), parathyroid gland (parathyroid glandular cells), bone, pituitary, adipose tissue	Magnesium and calcium homeostasis; regulation of cardiac function; cell viability (e.g., regulation of anoxic neuronal cell death); involvement in cytoskeletal organization, cell adhesion, cell migration and organogenesis.	Schlingmann et al., 2007 [138]; Zhang et al., 2012 [141]; Gwanyanya et al., 2021 [140]; Asrar and Aarts 2013 [142]; Turlova et al., 2021 [143]; Andriulė et al., 2021 [139]; Inoue et al., 2020 [144]
	TRPM8	2q37.2	Nerve ganglia (e.g., dorsal root ganglia, trigeminal ganglia) containing sensory neurons innervating the skin, oral cavity, lungs, bladder and prostate; Moreover, to varying degrees within colon, adipose tissue (including brown adipose tissue), liver, pancreas	Cold sensation and response to cold (cold-induced thermogenesis); modulation of pain sensation (pain relief or pain intensification, depending on the context and location); intracellular Ca^2+^ homeostasis; regulation of gastrointestinal motility	Qi et al., 2025 [145]; Ma et al., 2012 [146]; Moraes et al., 2017 [147]; Sun et al., 2021 [148]; Amato et al., 2020 [149]
TRPV (vanilloid)	TRPV1	17p13.3	Sensory neurons, immune cells, liver, pancreas, muscle cells, adipocytes, bladder, testis	Neuronal depolarization; temperature sensing; mechanosensing; pH-sensing; mediation of inflammatory pain and hyperalgesia	White et al., 2011 [150]; Aneiros et al., 2011 [151]; Xu et al., 2007 [152]; Shuba 2021 [153]; Li and Wang 2021 [154]
	TRPV2	17p11.2	Pulmonary and vein endothelial cells; nerve ganglia (e.g., dorsal root ganglia), spinal cord, brain, spleen, intestine	Temperature sensing; mechanosensing; regulation of immune response by augmenting B cell activation and function	Shibasaki 2016 [155]; Fricke and Leffler 2024 [156]; Liu and Qin 2016 [157]; Li et al., 2024 [158]
	TRPV3	17p13.3	Keratinocytes, corneal epithelial cells, nerve ganglia (e.g., dorsal root ganglia, trigeminal ganglia), spinal cord, brain, tongue, distal colon epithelium	Temperature sensing; vasoregulation	Lei and Tominaga 2025 [159]; Martin et al., 2024 [160]; Pires et al., 2015 [161]; Fromy et al., 2018 [162]
	TRPV4	12q24.11	Wide variety of cells/tissues including endothelial cells, nerve ganglia (e.g., dorsal root ganglia), skin, kidney, urinary bladder, lung, spleen, testis, keratinocytes, heart, liver, connective tissue (including adipocytes), bone marrow	Mechanosensing; temperature sensing and thermoregulation; immune response by regulating macrophage phagocytosis and cytokine production; ensuring cellular osmotic homeostasis	Hartmannsgruber et al., 2007 [163]; Güler et al., 2002 [164]; Baratchi et al., 2017 [165]; O’Neil and Heller 2005 [166]; Moore 2022 [167]; Orsini et al., 2024 [168]; Fukuda et al., 2024 [169]; Mamenko et al., 2015 [170]; Sánchez et al., 2016 [171]
	TRPV5	7q35	Kidney mainly (the distal convoluted tubules and connecting tubules), intestine, pancreas, placenta, brain	Modulation of calcium channel activity; calcium homeostasis by facilitating renal calcium reabsorption	Fluck et al., 2022 [172]; de Groot et al., 2009 [173]
	TRPV6	7q33–q34	Small intestine, pancreas, placenta, prostate, epididymis, kidney, salivary glands	A vitamin D-regulated Ca^2+^—selective channel required for Ca^2+^ homeostasis; maintenance of bone homeostasis and skeletal integrity; required for mammalian male fertility and maternal-fetal transport of Ca^2+^	Khattar et al., 2022 [174]; Bächinger et al., 2019 [175]; Lieben et al., 2010 [176]
TRPA (ankyrin)	TRPA1	8q13	Nerve ganglia (e.g., dorsal root ganglia, trigeminal ganglia), hair cells, ovary, spleen, testis, lung, heart, pancreas, liver, gastrointestinal tract, kidney, brain (including brain endothelial cells)	Mechanosensing; chemosensing (chemonociceptor); thermosensing; sensing of neuronal activity to regulate functional hyperemia and neurovascular coupling within the somatosensory cortex	Meents et al., 2019 [177]; Nielsen et al., 2018 [178]; Tominaga and Iwata 2025 [179]; Thakore et al., 2021 [180]
TRPP (polycystin)	TRPP2	4q21–q23	Widely expressed in vascular endothelial and smooth muscle cells of all major vascular beds, kidney	Mechanosensing Ca^2+^ channel in endothelial cells; regulation of intracellular Ca^2+^ homeostasis; regulation of vascular smooth muscle cell function towards optimizing contractility	Du et al., 2016 [181]; Sharif-Naeini et al., 2009 [182]; Gao et al., 2004 [183]
	TRPP3	10q24	Endothelial cells, kidney, heart, liver, spleen, tongue, retina, testis, neurons	Ca^2+^ influx in response to receptor stimulation; regulating vascular tone and permeability; contributing to endothelial cell hyperpolarization and vasodilation; angiogenesis and vascular remodeling; sour taste perception; sour sensitivity	Clapham 2003 [184]; Zheng et al., 2015 [185]; Lu et al., 2018 [186]; Zheng et al., 2016 [187]; Liu et al., 2023 [188]; Walsh et al., 2020 [189]
	TRPP5	5q31	Testis, heart, kidney	Ca^2+^ signaling; Ca^2+^ homeostasis in connection with cell proliferation or apoptosis; spermatogenesis;	Xiao et al., 2010 [190]; Chen et al., 2008 [191]
TRPML (mucolipin)	TRPML1	19p13.2–p13.3	Wide variety of tissues including brain, heart, skeletal muscle and endothelial cells	Ca^2+^ signaling and homeostasis of lysosomes (a lysosomal ion channel); endocytic and exocytic signaling events; immune response	Schmiege et al., 2017 [192]; Spix et al., 2020 [193]; Venkatachalam et al., 2015 [194]
	TRPML2	1p22	Lymphoid (A20 mature B lymphocyte, EL-4 T lymphocyte) and myeloid (5T33 myeloma) cell lines	B-lymphocyte development; endocytic and exocytic signaling events; immune response	Samie et al., 2009 [195]; Lindvall et al., 2005 [196]; Song et al., 2006 [197]; Spix et al., 2020 [193]
	TRPML3	1p22.3	Melanosomes of melanocytes; hair cells	Endocytic and exocytic signaling events; cell depolarization; overload with Ca^2+^; depending on the location of the hair cells: reception of acoustic stimuli (cochlea) or sensory element of the balance organ (semicircular canals and vestibule),	Di Palma et al., 2002 [198]; Grimm et al., 2007 [199]; Nagata et al., 2008 [200]; Atiba-Davies and Noben-Trauth 2007 [201]

**Table 2:** Involvement of PIEZO1 channel, transient receptor potential cation channel subfamily V member 3 (TRPV4) and receptor tyrosine kinases (RTKs) in mechanosensing/mechanotransduction at the trophoblast/placenta level based on their tissue expression [322,326,338,357]. Three categories of expression were distinguished: weak (+), moderate (++) and strong (+++).

Mechanoreceptor Type	Expression Level in Trophoblasts/Placental Cells				
	TSCs	EVTs	CTBs	STBs	FpEC
PIEZO1	++	+++	++	++	+++
TRPV4	+++	+	+++	+++	++
RTKs	+++	++	+++	+++	+++

## Data Availability

No new data were created. Instead, the data are quoted from the available cited literature.

## Abbreviations

4-AP	4-aminopyridine
7TM	seven transmembrane
aGPCRs	adhesion class of G protein-coupled receptors
Akt	protein kinase B (also known as PKB)
AQP3	aquaporin 3, a membrane transporter of water and glycerol
ARD	ankyrin repeats domains in TRPV4 channel
ASIC	acid-sensing ion channel
AT1R	angiotensin II type 1 receptor
AVPR1A	vasopressin V1a receptor
B2R	bradykinin receptor B2
CaM	calmodulin
CaPLSase	Ca^2+^-activated phospholipid scramblase
Cav	caveola
cGMP	cyclic guanosine monophosphate
CS	chondroitin sulfate
CSK	cytoskeleton
CTB	cytotrophoblast
D1BR	dopamine receptor D5
EC	endothelial cell
ECL1–ECL3	three extracellular loops 1–3
ECCJ	endothelial cell–cell junctions
ECM	extracellular matrix
EGFR	epidermal growth factor receptor (also known as ErbB)
ENaC/DEG	epithelial sodium channel/degenerin family
eNOS	endothelial nitric oxide synthase
enEVT	endovascular extravillous trophoblast (enEVT)
ER	endoplasmic reticulum
ERKs	extracellular signal-regulated kinases
ETB	endothelin B receptor
EVT	extravillous trophoblast
FAK	focal adhesion kinase
FAs	focal adhesions
FASL	Fas ligand (also known as CD95L or Apo-1L), a type-II transmembrane protein in the tumor necrosis factor (TNF) superfamily
FGR	fetal growth restriction
GAGs	glycosaminoglycans
GDP	guanosine diphosphate
GJ	gap junction
GLX	glycocalyx
GPCR	G protein-coupled receptor
GPR68	G protein-coupled receptor 68 (also known as OGR1)
GTP	guanosine triphosphate
H1R	histamine H1 receptor
HS	heparan sulfate
HSPGs	heparan sulfate proteoglycans
hTSCs	human trophoblast stem cells
HUGO	Human Genome Organization
ICAM-1	endothelial intercellular adhesion molecule-1
ICL1–ICL3	three intracellular loops 1–3
IEL	internal elastic lamina
IGF2BP1	insulin-like growth factor 2 mRNA-binding protein 1
IK_Ca_	intermediate conductance Ca^2+^-activated K^+^ channels
ILK	integrin-linked kinase
IP3	inositol trisphosphate
IP3R2	inositol 1,4,5-trisphosphate receptor type 2 (also known as ITPR2)
IUGR	intrauterine growth retardation
IUPHAR	International Union of Basic and Clinical Pharmacology
K2P	two-pore domain potassium channels
LINC	complex—linker of nucleoskeleton and cytoskeleton complex
MAPKs	mitogen-activated protein kinases
METTL14	protein methyltransferase-like 14
MSICs	mechanosensitive ion channels
NF-κB	nuclear factor kappa-light-chain-enhancer of activated B cells
NTF/CTF	complex—N-terminal fragment/C-terminal fragment complex of the adhesion class of G protein-coupled receptor
NO	nitric oxide
OS-9	lectin protein binding domain in TRPV4 channel
P1, P2	the two-pore domain K^+^ channels
PaO_2_	partial pressure of oxygen
PECAM-1	platelet endothelial adhesion molecule-1
PI3K	phosphatidylinositol 3-kinase
PKB	protein kinase B (also known as Akt)
PLC	phospholipase C
PRD	proline-rich domain in TRPV4 channel
ProGs	proteoglycans
pS	picosiemens
qRT-PCR	quantitative reverse transcription—polymerase chain reaction analysis
RTKs	receptor tyrosine kinases
S1PR1	sphingosine-1-phosphate receptor 1
sAC	soluble adenylyl cyclase
sGC	soluble guanylate cyclase
SI	International System of Units
STB	syncytiotrophoblast
SynT-2 STB	syncytiotrophoblast layer 2 cells
TALK	TWIK-related alkaline activated K^+^ channel
TASK	TWIK-related acid-sensitive K^+^ channels
TEA	tetraethylammonium
THIK	TWIK-related halothane inhibited K^+^ channel
TKs	non-receptor tyrosine kinases
TM	second transmembrane helix (transmembrane domain)
TMEM16F	transmembrane protein 16 F
TRAIL	tumor necrosis factor-related apoptosis-inducing ligand (also known as Apo2L)
TREK/TRAAK	TWIK-related K^+^ channels/TWIK-related arachidonic acid activated K+ channel
TRESK	TWIK-related spinal cord K^+^ channel
TRP	transient receptor potential channels
TRPA	transient receptor potential channel—ankyrin subfamily
TRPC	transient receptor potential channel—canonical subfamily
TRPM	transient receptor potential channel—melastatin subfamily
TRPML	transient receptor potential channel—mucolipin subfamily
TRPP	transient receptor potential channel—polycystin subfamily
TRPV	transient receptor potential channel—vanilloid subfamily
TSCs	trophoblast stem cells
TWIK	two-pore domain in a weak inward rectifying K^+^ channel
UtMVECs	uterine microvascular endothelial cells
VE-cadherin	vascular endothelial cadherin (also known as CDH5)
VEGFR2, VEGFR3	vascular endothelial growth factor receptors 2 and 3
VSMC	vascular smooth muscle cells
